# Toenail Chromium Concentration and Metabolic Syndrome among Korean Adults

**DOI:** 10.3390/ijerph15040682

**Published:** 2018-04-04

**Authors:** Jakyung Son, J. Steven Morris, Kyong Park

**Affiliations:** 1 Department of Food and Nutrition, Yeungnam University, Gyeongsan, Gyeongbuk 38541, Korea; jakyungson@ynu.ac.kr; 2 Department of Research and Education, University of Missouri Research Reactor, Columbia, MO 65211, USA; morrisj@missouri.edu; 3 Department of Research Services, Harry S. Truman Memorial Veterans Hospital, Columbia, MO 65205, USA

**Keywords:** chromium, metabolic syndrome X, nails, biomarkers, Asia

## Abstract

Although in vivo and in vitro studies have shown that chromium has protective effects against metabolic diseases, few studies have examined this association in humans. The present study examined chronic chromium (Cr) exposure among Koreans based on the measurement of toenail Cr concentrations, and analyzed the associations between toenail Cr concentrations and metabolic syndrome (MetS) and its components. We conducted a cross-sectional analysis using baseline data from the prospective cohort study in the Yeungnam area of South Korea that included 232 men and 268 women. Toenail Cr concentration was quantified by neutron activation analysis, and metabolic biomarker levels were obtained through medical examinations. The odd ratios (OR) of prevalent MetS and its components in correlation with Cr concentrations were calculated using multivariable logistic regression. After multiple confounding variables were adjusted for, participants with higher concentrations of Cr had a prevalence rate of MetS similar to those with lower concentrations (OR, 1.84; 95% confidence interval, 0.65–5.23). Our results do not support an association between long-term exposure to Cr and a lower prevalence of MetS in Koreans, whose Cr concentrations are relatively low compared to those of populations in Europe and the United States.

## 1. Introduction

Chromium (Cr) is a trace nutrient that plays a role in carbohydrate and lipid metabolism in the human body [1]. Recent meta-analysis results have confirmed the beneficial effects of Cr, showing that Cr supplementation significantly improved glycemic control and decreased the risk for diabetes mellitus [2,3]. Although metabolic syndrome (MetS) and diabetes mellitus share multiple biological pathways, few studies have examined the association between Cr exposure and MetS, a combination of risk factors for diabetes mellitus and cardiovascular diseases. One study, conducted by Bai et al., reported that toenail Cr concentrations were inversely associated with the incidence of MetS in young American adults whose baseline median toenail Cr concentrations ranged from 0.2 (first quartile) to 3.5 μg/g (fourth quartile) [4]. However, no study has examined this association in an Asian population.

The results reported from Western countries regarding the effects of Cr exposure on health conditions cannot be directly generalized to Asian populations because Cr exposure range, affected by local soil Cr concentrations, can vary across different geographic areas [5]; thus, the results from the relative comparisons within Western populations may not be comparable to those of Asians.

Since MetS has become a significant public health burden in Asia [6,7], it is crucial to identify independent dietary factors of MetS, including Cr. Therefore, the aims of the present study were to examine chronic Cr exposure among Koreans based on the measurement of toenail Cr concentrations, and to analyze the associations between toenail Cr concentrations and MetS and its components using baseline data from a community based cohort study.

## 2. Materials and Methods

### 2.1. Study Population

This study analyzed the baseline data of the trace element study of Korean adults in the Yeungnam area (SELEN), an ongoing prospective cohort study in South Korea [8]. Baseline data of community-dwelling individuals aged 35 years or older residing in the southeastern region of South Korea was collected from December 2012 to December 2013. This analysis included 500 individuals (232 men and 268 women) with complete datasets of their general characteristics, health examination findings, as well as toenail Cr concentrations.

### 2.2. Ethical Statement

The SELEN cohort study was approved by the Institutional Review Board of Yeungnam University Medical Centre (IRB number: YUH-12-0468-O94, 7002016-A-2015-006) and adhered to the tenets of the Declaration of Helsinki. All participants provided informed consent.

### 2.3. Demographic and Personal Behavior Information

The general characteristics of the participants in this study, including demographic information and personal behavior were collected through self-administered questionnaires. Education levels were classified into two groups (high school graduate or less and college graduate or higher). Monthly household income was categorized into four groups (<3,000,000; 3,000,000–4,000,000; 4,000,000–6,000,000, and ≥6,000,000 Korean Republic won). The alcohol consumption status was categorized as current drinker or non-drinker. Physical activity levels were determined using metabolic equivalent tasks (METs; h/week), calculating time in vigorous and moderate activities and walking with weighted duration and frequency of activities [9].

### 2.4. Dietary and Health Information

Usual diet was assessed using a validated 146-item, semi-quantitative food frequency questionnaire (SQFFQ) [10]. Nutrient intake levels, including total energy intake, were calculated using Computer Aided Nutritional analysis program (CAN-Pro, version 4.0; The Korean Science and Technology Center, Seoul, South Korea).

Participants’ health information was collected through regular medical examinations conducted by the Korean National Health Insurance Service [11] or medical institutions. Participants were asked to fast for at least 12 h before undergoing the examinations. Anthropometric data, including height, weight, and waist circumference, were measured by trained nurses at the clinic. Fasting blood glucose, triglyceride (TG), high-density lipoprotein (HDL) cholesterol were measured at the clinical laboratories in cooperation with the Korean National Health Insurance Service [11]. MetS was defined according to the modified National Cholesterol Education Program III for Asians criteria [12]. Participants with at least three of the following criteria were defined as having MetS:

1. Fasting glucose ≥100 mg/dL or medication use (insulin or oral agents);
2. Blood pressure ≥130/85 mmHg or antihypertension medication use;
3. Waist circumferences ≥90 cm in men and ≥80 cm in women;
4. TG ≥ 150 mg/dL; and
5. HDL cholesterol <40 mg/dL in men and <50 mg/dL in women, or medication use.

### 2.5. Toenail Cr

The SELEN study collected toenail clippings from participants in order to measure long-term exposure to trace elements, including Cr. After collecting toenail clippings from all 10 toes of each participant, the SELEN investigators inspected the samples for contamination (manicured nails or nails dyed with garden balsam). Next, the samples were shipped to the University of Missouri Research Reactor (Columbia, MO, USA) for analysis of toenail Cr concentrations using neutron activation analysis (NAA). Details of the analytic methods and NAA validation have been published elsewhere [13]. Specifically, toenail samples were grouped (with quality control samples, comparison standards, and analytical blanks, which were also prepared in vacuum sealed quartz vials) into two irradiation packages each containing a dilute cobalt in aluminum neutron-density monitor. Each bundle contained approximately 40 samples, six standards, six quality control samples, and three analytical blanks. Two bundles were placed in aluminum cans closed with welded lids and irradiated for 50 h in a rotated irradiation position in the graphite reflector of the 10 megawatt, steady state, research reactor at the University of Missouri Research Reactor (MURR) Centre in a typical thermal flux of 6.33 ± 0.11 n/cm^2^/s (bundle 1) and 5.40 ± 0.17 n/cm^2^/s (bundle 2) as determined by the neutron monitors. The irradiated toenail samples, quality-control samples, standards and blanks were allowed to decay for approximately four days. Then the sealed vials were cleaned overnight in freshly-prepared aqua-regia. The cleaned vials were then washed with copious volumes of water, dried and placed in plastic counting tubes and were analyzed by high-resolution gamma-ray spectroscopy using a high-purity germanium detector (ORTEC, Oak Ridge, TN, USA) coupled to a Lynx Digital Signal Analyzer operated through Genie software (Canberra Industries, Inc., Meriden, CT, USA) and coupled to the MURR GRS Network. Samples were introduced to the auto-rotated measurement position using a shielded automated sample changer designed and constructed at the MURR Centre. Each toenail sample quality control sample, standard and blank was individually transferred into the auto-rotated counting position and measured for 7200 s live time. Pulse-pileup corrections were done using the internal Lynx Digital Signal Analyzer software. The spectrometer components (hardware and software) were checked weekly and were in proper calibration during the measurement periods used for this project. The spectral data are collected in 8196 storage channels over an approximate energy range of 50 to 3000 keV. Each spectrum is stored in a designated project file on the MURR Centre’s GRS Network with a redundant backup. Chromium concentrations were determined by standard comparison. Chromium comparison standards were prepared from a certified standard solution provided by High-Purity Standards, Charleston, SC, USA, and traceable to the U.S. National Institutes of Standards and Technology (NIST), Gaithersburg, MD, USA. A biological standard reference material (SRM 1571 Orchard Leaves) obtained from NIST was analyzed with each batch of samples. The mean ± SD chromium concentration measured in this SRM, 2.59 ± 0.19 µg/g is in good agreement with the certified value, 2.6 ± 0.3 µg/g.

### 2.6. Statistical Analysis

We conducted a sample size calculation using G*Power software 3.1 with an α-error probability of 0.05, power (1-β error probability) of 0.80, probability of 0.17 [14], and effect size (odds ratio, OR) of 1.62 [15]. Based on the result of this calculation, the total sample size needed was at least 246. Assuming 30% of the data would be missing due to attrition or measurement errors, we determined the necessary total sample size to be approximately 500 participants.

The distributions of continuous variables were examined and logarithmically transformed if the distribution was skewed to the right. The age- and sex-adjusted means of fasting serum biomarkers, blood pressure, anthropometry, and blood lipids were compared across the quartiles of toenail Cr concentration. The *p* values for trends were calculated using the median value of the category of toenail Cr in generalized linear regression analysis. Multiple potential confounding variables and effect modifiers, such as age, sex, total energy intake, smoking status, alcohol consumption, monthly household income, physical activity, and toenail levels of selenium and mercury were determined based on previous studies [4,16] and preliminary analysis. We found no effect modifier in the association between toenail Cr and MetS. The current study evaluated three covariate models using logistic regression: model 1 was adjusted for age, sex, and total energy intake; model 2 was additionally adjusted for smoking status, alcohol consumption, monthly household income, and physical activity; and model 3 was further adjusted for toenail levels of selenium and mercury. All statistical analysis in this study used Statistical Analysis System (SAS, version 9.4; SAS Institute, Inc., Cary, NC, USA), and the two-tailed, critical value for *p* was set at α = 0.05.

## 3. Results

### 3.1. General Characteristics

Study participants were categorized into quartile according to their toenail Cr concentrations, and the general characteristics of the study participants were compared among the groups (Table 1). The median levels of toenail Cr concentrations were 0.06, 0.13, 0.20, and 0.49 μg/g in each group, respectively. The mean age of the participants was approximately 44 years, and approximately half of the participants in each group were men. The mean age and sex proportions did not differ among the groups. The proportions of college graduates or higher were similar among the four groups (*p* = 0.1).

### 3.2. Metabolic Biomarkers

In Table 2, age- and sex-adjusted measurements of fasting serum biomarkers, blood pressure, anthropometry, and blood lipids are compared among the categories of toenail Cr concentration. The means of fasting blood glucose, systolic and diastolic blood pressures, waist circumference, TG, and HDL cholesterol were not significantly different among the quartiles of toenail Cr concentrations.

### 3.3. Metabolic Syndrome and Its Components

Table 3 shows the ORs and 95% confidence intervals (CIs) of MetS and its components according to the categories of toenail Cr concentrations based on logistic regression models adjusted for potential confounding variables in three different models. In the fully-adjusted models, the associations between toenail Cr concentrations and prevalent metabolic health outcomes were not statistically significant, showing MetS (OR, 1.84; 95% CI, 0.65–5.23), high fasting glucose (OR, 1.51; 95% CI, 0.71–3.18), high blood pressure (OR, 1.23; 95% CI, 0.67–2.25), abdominal obesity (OR, 0.89; 95% CI, 0.43–1.84), hypertriglyceridemia (OR, 0.97; 95% CI, 0.49–1.92), hypo-HDL cholesterolemia (OR, 1.37; 95% CI, 0.68–2.78), and in the highest toenail Cr concentration compared to the lowest toenail Cr concentration.

## 4. Discussion

Median concentrations of the lowest and highest groups of toenail Cr among study participants were 0.06 μg/g and 0.49 μg/g, respectively. These were very low concentrations when compared with toenail Cr concentrations reported in Western populations by previous studies [4,16,17,18,19,20]. In the current study, there was no significant association between the toenail Cr concentration and the prevalence of MetS and its components.

Cr exposure can be measured using various biomarkers in the blood (whole blood, serum, and erythrocytes), urine, hair, and nails. Blood and urine reflect relatively short term mineral exposure [13]. These are mostly used to measure the degree of acute Cr exposure among workers in industrial sites who are likely to be exposed to Cr in their work environments [21,22]. Hair Cr has a limitation of contamination by external materials, such as shampoos and hair dyes [23]. Fingernails and toenails have been reported to be useful for examining long-term trace element exposure [24]. Although fingernail Cr concentrations are more likely to be contaminated by external materials, such as nail polishes and hand cream, toenail Cr concentrations are less commonly contaminated and, therefore, may be the most useful for assessing long-term Cr exposure [24]. In addition, the measurement of trace elements in toenail clippings has been shown to be highly reproducible [13].

Several prior studies also examined toenail Cr concentrations using NAA at the University of Missouri Research Reactor. Coronary Artery Risk Development in Young Adults (CARDIA) is an ongoing cohort study that follows up with young adult men and women living in four major cities in the United States (US). The median baseline toenail Cr concentration in the CARDIA study was 1.2 μg/g, which is about 7.5 times that of the SELEN participants in the present study [4]. The Nurses’ Health Study (NHS) and the Health Professionals Follow-up Study (HPFS) also reported toenail Cr concentrations measured with NAA at the University of Missouri Research Reactor. The median value among the NHS participants was 1.2 μg/g [17], a concentration similar to that of the CARDIA participants. The HPFS study found median values ranging from 0.23 μg/g (first quartile) to 2.08 μg/g (fourth quartile) [16], which are much higher than the toenail Cr concentrations of the SELEN participants in the current study.

European studies have also reported much higher toenail Cr concentrations compared with participants of the present study. The median toenail Cr concentration was 0.5 μg/g for adults in Ireland [18], 1.30 μg/g among men living in 10 European regions, including Finland, Germany, and Scotland, as well as Israel [19], and 1.13 μg/g for Iranians [20]. A comparison of the Cr exposure levels in the current study with those conducted in Western countries revealed substantially lower concentrations among the Korean participants and a narrower range of exposure.

The positive effect of Cr on glucose metabolism was first reported by Glinsmann et al. [25] and, since then, Cr has been found to play an essential role in energy metabolism (glucose, fat, and protein) by increasing insulin activity [1,5]. Furthermore, Cr supplements have been reported to improve glucose intolerance in patients with diabetes, and influence carbohydrate and lipid metabolism by interacting with insulin [25]. Recently, Yin et al. conducted a meta-analysis of 14 randomized controlled trials (RCTs) and cohort studies in order to elucidate the association between Cr and chronic diseases [2]. In this meta-analysis, patients with diabetes who had ingested a Cr supplement (Brewer’s yeast) showed significantly lower fasting blood sugar (lower by 19.23 mg/dL; 95% CI, −35.30 to −3.16) compared to those of the control group. Another meta-analysis examined 25 RCTs of patients with diabetes who had ingested Cr supplements, and the authors reported that their glycated haemoglobin were lower by 0.55% (95% CI, −0.88 to −0.22) than those of the control group; their fasting blood sugar were also lower by 20.72 mg/dL (95% CI, −1.84 to −0.47) than those of the control group [3]. A meta-analysis of 20 RCTs of overweight or obese participants who had ingested Cr supplements showed that their weight was lower by 0.50 kg (95% CI, −0.97 to −0.03) than the weight of participants who had not received Cr supplementation [26]. These results support a beneficial effect from Cr supplements.

A recent study in the US analyzed the association between toenail Cr concentration and the incidence of MetS. When the CARDIA subjects were classified into four groups according to toenail Cr concentration and these groups were compared, the risk of MetS in the group with the highest concentrations was approximately 20% lower than the risk for the lowest concentration group (Q4 vs. Q1: hazard ratio, 0.8; 95% CI, 0.66–0.98; *p* for trend = 0.006) [4]. In the current study, however, no association was found between toenail Cr concentration and the prevalence of various cardiovascular disorders, including MetS and glucose metabolic abnormalities. This is probably because the toenail Cr concentration of the participants in the SELEN study was 3–10 times lower than that measured in studies in other regions (America, Europe, and Asia). The range of exposure was also very narrow in the SELEN study population, resulting in a non-significant difference in prevalent metabolic conditions based on the toenail Cr concentrations observed.

There are several limitations in this study. We collected metabolic biomarker information (main outcomes) only six months after completion of the exposure variable data collection; thus, we analyzed data in a cross-sectional manner, and causal inferences cannot be made. However, toenail Cr concentration reflects long term exposure of up to one year; therefore, the use of chromium as a biomarker may have reduced the occurrence of reverse causation. Another limitation is the small number of cases of metabolic syndrome, leading to low statistical power to detect any association between toenail chromium and metabolic syndrome. In addition, there may be residual confounding effects, although we considered potential multiple confounding factors, such as demographic characteristics, and lifestyle and family history, in the analysis. Furthermore, the self-administered questionnaire and a single measurement of toenail Cr may have resulted in random error with underestimated results.

Despite these disadvantages, this study has several strengths. First, this is the first study to examine the association between chronic Cr exposure and MetS in Asians. Therefore, the results provide meaningful scientific data for the revision of the dietary guidelines on Cr in Koreans. Second, we used toenail Cr concentration as a reliable biomarker to reflect relatively long-term exposure and a valid method to analyze the concentration of toenail Cr. 

## 5. Conclusions

The Cr exposure of the Koreans studied was very low, and no significant cross-sectional association was observed between Cr and MetS within the observed range of Cr exposures. In the future, the effects of Cr supplementation in Korean patients with diabetes need to be investigated in large-scale cohort studies and various RCTs.

## Figures and Tables

**Table 1:** Characteristics of participants according to quartiles of toenail chromium concentrations, the SELEN study (*n* = 500).

Characteristics	Toenail Chromium Concentrations				*p* Value ^1^
	Q1 (Lowest)	Q2	Q3	Q4 (Highest)	
No. of participants	125	125	125	125	
Range (median), μg/g	0.003–0.10 (0.06)	0.11–0.16 (0.13)	0.17–0.27 (0.20)	0.28–5.76 (0.49)	
Age, years	45.1 ± 5.5	44.3 ± 5.2	45.3 ± 5.5	44.5 ± 5.2	0.6
Women	52.0	52.0	55.2	55.2	0.9
Current smokers	20.8	20.8	19.2	14.4	0.5
Current drinkers	81.6	80.0	70.4	82.4	0.1
Physical activity, MET-h/week	35.4 ± 51.2	34.5 ± 46.2	27.3 ± 41.5	34.7 ± 46.4	0.9
Education					0.1
High school graduation or less	5.6	2.4	4.8	0.8	
College graduation or higher	94.4	97.6	95.2	99.2	
Monthly household income, KRW					0.9
<3,000,000	24.0	21.6	20.8	19.2	
3,000,000–<4,000,000	27.2	20.8	23.2	26.4	
4,000,000–<6,000,000	32.0	34.4	32.0	33.6	
≥6,000,000	16.8	23.2	24.0	20.8	
Total energy intake, kcal/day	1871 ± 651	1755 ± 521	1847 ± 541	1800 ± 544	0.6
Toenail minerals, μg/g					
Selenium	0.69 ± 0.09	0.69 ± 0.09	0.68 ± 0.09	0.70 ± 0.08	0.6
Mercury	0.41 ± 0.25	0.39 ± 0.22	0.39 ± 0.26	0.43 ± 0.31	0.3

Values are mean ± standard deviation or %. Abbreviations: SELEN, Trace Element Study of Korea Adults in the Yeungnam area; Q, quartile; METs, metabolic equivalent tasks; KRW, Korean Republic Won. ^1^ *p* values are derived from chi-squared test for categorical variables and *p* for trends across quartiles of toenail chromium concentrations was calculated using generalized linear models.

**Table 2:** Metabolic biomarkers of participants according to quartiles of toenail chromium concentrations, the SELEN study (*n* = 500).

Metabolic Biomarkers	Toenail Chromium Concentrations				*p* for Trend ^1^
	Q1 (Lowest)	Q2	Q3	Q4 (Highest)	
No. of participants	125	125	125	125	
Fasting blood glucose, mg/dL	90.9 ± 1.1	90.8 ± 1.1	92.3 ± 1.1	92.5 ± 1.1	0.2
Systolic blood pressure, mmHg	117.7 ± 1.2	116.5 ± 1.2	117.9 ± 1.2	118.2 ± 1.2	0.5
Diastolic blood pressure, mmHg	73.3 ± 0.9	73.7 ± 0.9	72.5 ± 0.9	74.2 ± 0.9	0.4
Waist circumference, cm	78.6 ± 0.7	77.5 ± 0.8	78.8 ± 0.7	78.2 ± 0.8	0.9
Triglyceride, mg/dL	108.4 ± 6.0	116.0 ± 6.0	118.8 ± 6.0	116.6 ± 6.0	0.5
HDL cholesterol, mg/dL	118.7 ± 2.5	114.1 ± 2.5	117.8 ± 2.5	114.4 ± 2.5	0.4

Values are age- and sex-adjusted means ± standard error. Abbreviations: SELEN, Trace Element Study of Korea Adults in the Yeungnam area; Q, quartile; HDL, high-density lipoprotein. ^1^ *p* for trend across quartiles of processed meat intake was calculated using generalized linear models.

**Table 3:** Odds ratio and 95% confidence interval for metabolic syndrome and its components in participants according to quartiles of toenail chromium concentrations, the SELEN study (*n* = 500).

	Toenail Chromium Concentrations				*p* for Trend ^1^
	Q1 (Lowest)	Q2	Q3	Q4 (Highest)	
No. of participants	125	125	125	125	
Range (median), μg/g	0.003–0.10 (0.06)	0.11–0.16 (0.13)	0.17–0.27 (0.20)	0.28–5.76 (0.49)	
High fasting glucose					
Case, *n* (%)	18 (14.4)	19 (15.2)	24 (19.2)	22 (17.6)	
Model 1	1	1.06 (0.52–2.16)	1.44 (0.72–2.85)	1.28 (0.64–2.56)	0.5
Model 2	1	1.34 (0.63–2.85)	1.93 (0.93–4.02)	1.52 (0.72–3.18)	0.4
Model 3	1	1.35 (0.63–2.91)	1.98 (0.94–4.15)	1.51 (0.71–3.18)	0.4
High blood pressure					
Case, *n* (%)	29 (23.2)	29 (23.2)	31 (24.8)	34 (27.2)	
Model 1	1	1.04 (0.57–1.90)	1.11 (0.61–2.00)	1.32 (0.74–2.37)	0.3
Model 2	1	1.06 (0.57–1.96)	1.17 (0.64–2.15)	1.28 (0.70–2.32)	0.4
Model 3	1	1.01 (0.54–1.88)	1.14 (0.61–2.11)	1.23 (0.67–2.25)	0.5
Abdominal obesity					
Case, *n* (%)	18 (14.4)	17 (13.6)	24 (19.2)	18 (14.4)	
Model 1	1	0.93 (0.45–1.91)	1.37 (0.70–2.69)	0.99 (0.48–2.01)	0.9
Model 2	1	0.91 (0.44–1.88)	1.29 (0.65–2.56)	0.96 (0.47–1.96)	0.9
Model 3	1	0.87 (0.42–1.83)	1.24 (0.62–2.48)	0.89 (0.43–1.84)	0.8
Hypertriglyceridemia					
Case, *n* (%)	25 (20.0)	30 (24.0)	21 (16.8)	24 (19.2)	
Model 1	1	1.25 (0.67–2.34)	0.82 (0.42–1.58)	0.96 (0.50–1.83)	0.7
Model 2	1	1.37 (0.72–2.63)	0.84 (0.42–1.66)	1.05 (0.54–2.04)	0.8
Model 3	1	1.48 (0.76–2.87)	0.82 (0.41–1.66)	0.97 (0.49–1.92)	0.6
Hypo-HDL cholesterolemia					
Case, *n* (%)	19 (15.2)	24 (19.2)	23 (18.4)	24 (19.2)	
Model 1	1	1.31 (0.67–2.58)	1.20 (0.61–2.37)	1.26 (0.64–2.47)	0.7
Model 2	1	1.31 (0.65–2.63)	1.13 (0.56–2.27)	1.32 (0.66–2.64)	0.6
Model 3	1	1.31 (0.65–2.66)	1.18 (0.58–2.38)	1.37 (0.68–2.78)	0.5
Metabolic syndrome					
Case, *n* (%)	5 (4.0)	11 (8.8)	9 (7.2)	13 (10.4)	
Model 1	1	2.05 (0.74–5.68)	1.46 (0.50–4.26)	2.21 (0.81–6.07)	0.2
Model 2	1	2.14 (0.76–6.02)	1.39 (0.47–4.11)	2.17 (0.78–6.04)	0.3
Model 3	1	2.07 (0.73–5.90)	1.29 (0.43–3.90)	1.84 (0.65–5.23)	0.5

Model 1: Adjusted for age, sex, and total energy intake. Model 2: Additionally adjusted for smoking status, alcohol consumption, monthly household income, and physical activity. Model 3: Additionally adjusted for toenail concentrations of selenium and mercury. Abbreviations: SELEN, Trace Element Study of Korea Adults in the Yeungnam area; Q, quartile; HDL, high-density lipoprotein. ^1^ *p* for trend across quartiles of toenail chromium concentrations was calculated using generalized linear models.
